# Multi-Layer SnSe Nanoflake Field-Effect Transistors with Low-Resistance Au Ohmic Contacts

**DOI:** 10.1186/s11671-017-2145-2

**Published:** 2017-05-25

**Authors:** Sang-Hyeok Cho, Kwanghee Cho, No-Won Park, Soonyong Park, Jung-Hyuk Koh, Sang-Kwon Lee

**Affiliations:** 1 Department of Physics, Chung-Ang University, Seoul, 06974 Republic of Korea; 2 School of Electrical and Electronics Engineering, Chung-Ang University, Seoul, 06974 Republic of Korea

**Keywords:** Tin chalcogenides, Tin monoselenide (SnSe), Carrier screening effect, Field-effect transistors, 2-D materials, Metal work function

## Abstract

We report p-type tin monoselenide (SnSe) single crystals, grown in double-sealed quartz ampoules using a modified Bridgman technique at 920 °C. X-ray powder diffraction (XRD) and energy dispersive X-ray spectroscopy (EDX) measurements clearly confirm that the grown SnSe consists of single-crystal SnSe. Electrical transport of multi-layer SnSe nanoflakes, which were prepared by exfoliation from bulk single crystals, was conducted using back-gated field-effect transistor (FET) structures with Au and Ti contacts on SiO_2_/Si substrates, revealing that multi-layer SnSe nanoflakes exhibit p-type semiconductor characteristics owing to the Sn vacancies on the surfaces of SnSe nanoflakes. In addition, a strong carrier screening effect was observed in 70−90-nm-thick SnSe nanoflake FETs. Furthermore, the effect of the metal contacts to multi-layer SnSe nanoflake-based FETs is also discussed with two different metals, such as Ti/Au and Au contacts.

## Background

Transient metal chalcogenides offer a range of optical bandgaps, which make these materials suitable for use in various optical and optoelectronic applications [1]. Thin films of these materials, including PbTe, PbSe, and Bi_2_Se_3_ [2], have attracted considerable attention owing to their prospective usage in infrared optoelectronics devices, radiation detectors, solar cells, memory devices, and holographic recording devices [3–8]. Tin mono and diselenides (SnSe and SnSe_2_) have been in the limelight of research, owing to their high absorption coefficients, which is advantageous for optoelectronic applications. In addition, these materials are promising for use in thermoelectric applications [9–24]. Tin monoselenide (SnSe) is a p-type semiconductor with a bandgap for indirect allowed transitions close to ~0.9 eV and that for direct allowed transitions close to ~1.2 eV, whereas tin diselenide (SnSe_2_) is an n-type semiconductor [6]. The crystal structure of SnSe is orthorhombic, and its unit cell parameters are *a* = 11.496 Å, *b* = 4.151 Å, and *c* = 4.444 Å; this orthorhombic structure transforms into a tetragonal structure at high temperature that is nevertheless lower than the melting point of SnSe_2_ [25].

Recently, Sn-based binary chalcogenide and dichalcogenide electrical devices, including field-effect transistors (FETs) with a large-area common back gate, have been extensively investigated. In particular, much progress has been made in characterizing Sn dichalcogenide-based FETs [26, 27]. In 2016, Pei et al*.* reported a few-layer SnSe_2_ FET, demonstrating a high on/off ratio of ~10^4^ with a top capping layer of a polymer electrolyte [27]. Guo et al. also reported a high-mobility few-layer SnSe_2_ FET with a thickness of ~8.6 nm [28]. From previous works, it was confirmed that thin and low-carrier concentration SnSe films yield high mobility and current on/off ratio of SnSe_2_ FETs. Despite these efforts in utilizing SnSe_2_, however, electrical characterization of SnSe FETs, prepared by exfoliation from single crystals, has not been reported. A detailed characterization of electrical transport in few- and multi-layer SnSe_2_ flakes has to be performed for assessing the electrical transport properties of tin chalcogenides, such as SnSe nanoflakes, because single SnSe crystals are expected to exhibit a high carrier mobility of ~7835 cm^2^/V s [29].

In this work, we characterized single crystalline SnSe grown by using a modified Bridgman method. Electrical transport in multi-layer SnSe nanoflake FETs prepared by exfoliation from bulk single crystals was characterized for the first time using back-gated FET structures on SiO_2_/Si substrates. Furthermore, the effect of metal contacts on multi-layer SnSe nanoflake-based FETs was also studied for two different types of contacts (Ti/Au and Au) because the contact metal’s work function determines the conduction of hole carriers through the Schottky barrier at the metal-SnSe nanoflake interface.

## Methods

**Fig. 1:**
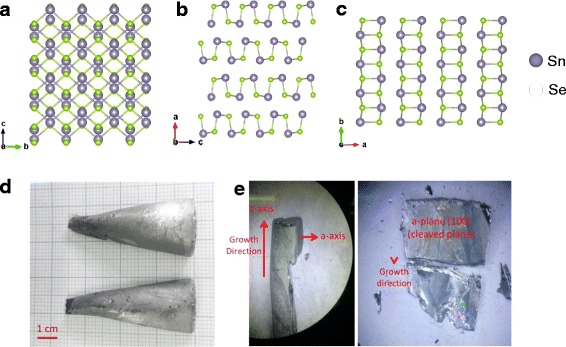
**a**–**c** Crystal structure of SnSe crystals along *a*-, *b*-, and *c*-axial directions. **d** Photograph of a grown single-crystalline SnSe. **e** Photographs of cleaved SnSe crystals along the *a*-axis (100) plane. Top views of the cleaved plane (100) of the SnSe single crystal (*right image* in **d**)

SnSe has a layered orthorhombic crystal structure at room temperature [24]. Figure 1a–c shows the perspective views of the SnSe crystal structure along the *a*, *b*, and *c* axial directions. As shown in Fig. 1c, SnSe slabs with two-atom layer are grooved, whereas easy cleavage in the crystals occurs along the (100) plane (Fig. 1b). Single SnSe crystals were grown by using the modified Bridgman method, as described previously [24, 30]. Stoichiometric amounts of Sn (99.999% shot, Alfa Aesar) and Se (99.999% powder, Alfa Aesar) were first melted to an ingot (~20 g) in a double-sealed quartz ampoule. The raw materials were slowly heated to 500 °C and dwelled for 10 h, then held at 920 °C for additional 10 h before shutting off the furnace. The obtained ingot was ground into powder and filled in a cone-shaped quartz tube, evacuated, and flame-sealed. This charged cone-shaped quartz tube was placed into a larger quartz tube. The outer tube was filled with gaseous Ar for preventing explosion and oxidation, and then flame-sealed. The charged quartz ampoule was placed at the position at which the temperature gradient was the largest in the vertical tube furnace. The vertical tube furnace was slowly heated to 970 °C for 20 h, held for 10 h, and then cooled down to 830 °C at the rate of 0.5 °C/h. The furnace was held at 830 °C for additional 24 h and then cooled down to 500 °C at the rate of 100 °C /h before shutting off the furnace.

## Results and Discussion

**Fig. 2:**
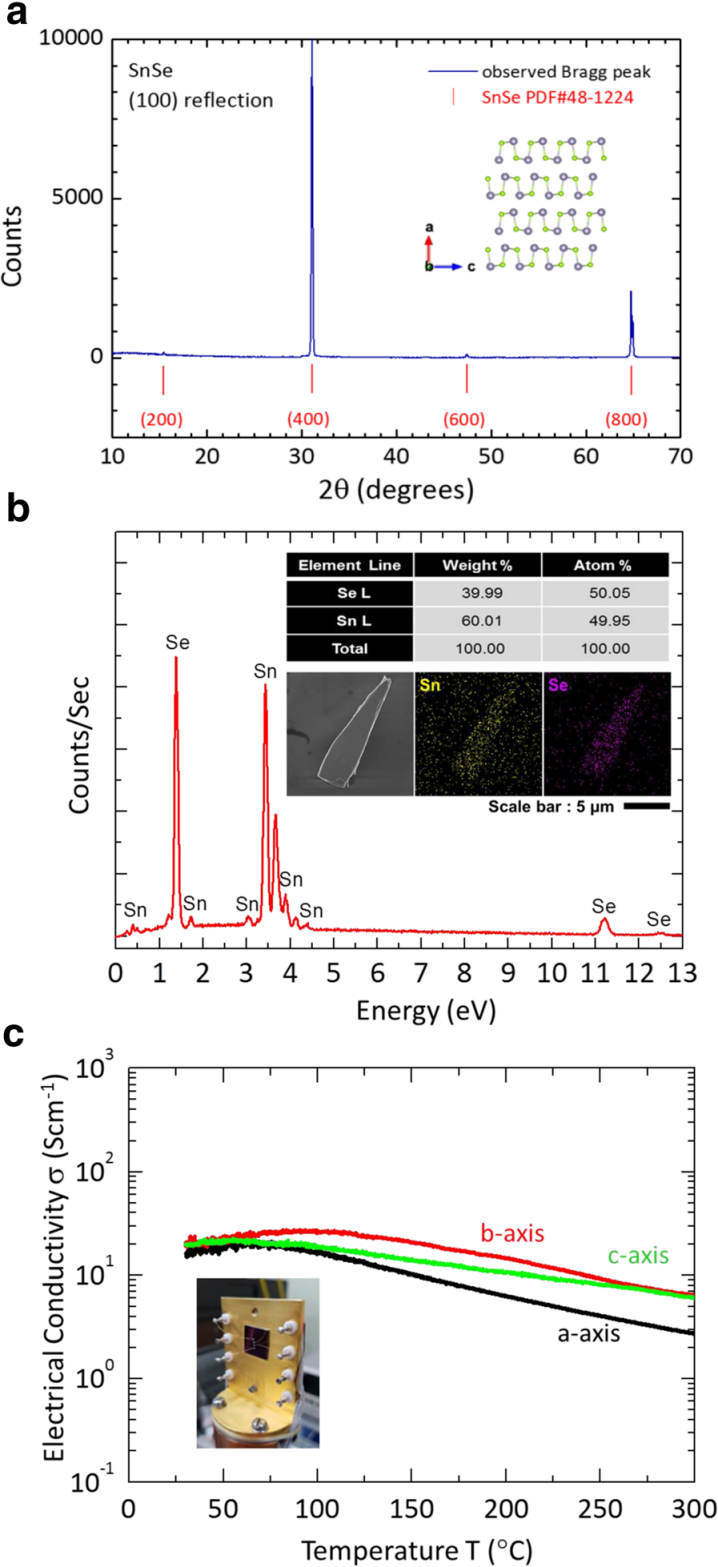
**a** XRD pattern of SnSe single crystals, showing a clear (h00) diffraction. **b** EDX spectrum of the SnSe single crystals*. Inset* shows the detailed information of atomic ratio of Sn and Se. *Insets* exhibit a SEM image and EDX mapping of a SnSe nanoflake FET with a device thickness of 90 nm. **c** Temperature-dependent electrical conductivity of the SnSe single crystals, for temperatures ranging from 30–300 K, measured using the four-point-probe method

A cone-shaped SnSe crystal (diameter, 30 mm; length, 70 mm) was obtained and is shown in Fig. 1d–e. The obtained crystal was divided into two pieces owing to a crack that occurred during the crystal’s extraction from the quartz ampoule (Fig. 1d). The quality of the grown SnSe crystals was checked by using a powder X-ray diffractometer (XRD, New D8-Advance, Bruker-AXS, Germany) with Cu *Kα* (*λ* = 1.5406 Å). Figure 2a shows the XRD pattern of the power diffraction file (PDF) 48–1224 for orthorhombic SnSe, together with the pattern for the crystallographic *a* axis, which is perpendicular to the cleaved plane (100) of the SnSe crystal. As shown in Fig. 2a, the XRD pattern of single crystalline SnSe strongly suggests a single-phase orthorhombic crystal with a space group *Pnma* [31], indicating a strong preferred orientation with (h00) reflections, which is in good agreement with a previous report [32]. In addition, the predominant peaks were (400) and (800), at 2*θ =* 31.081° and 64.818°, respectively, as shown in Fig. 2a [33]. As shown in Fig. 2b, energy dispersive X-ray spectroscopy (EDX) revealed the Sn:Se atomic ratio of 1:1, confirming the stoichiometric ratio of Sn and Se (inset of Fig. 2b). Insets of Fig. 2b also show a scanning electron microscopy (SEM) image and EDX mapping of a SnSe nanoflake FET with a device thickness of 90 nm. This result is in a good agreement with previous reports [24, 33]. In addition, the electrical conductivity of single crystalline SnSe (inset of Fig. 2b) was measured for temperatures ranging from 30‒300 K, using the conventional four-point-probe method. Figure 2c shows the temperature-dependent electrical conductivity of SnSe single crystals along three different crystallographic directions, indicating similar temperature-dependent behaviors and anisotropy behaviors owing to different hole mobilities in SnSe along the different axial directions. As shown in Fig. 2c, the electrical conductivity along the *b*- and *c*-axis at 300 K was determined to be ~6.00 S cm^−1^, which is ~2.2 times larger than that for the *a*-axis (~2.7 S cm^−1^). This result is in a good agreement with previous results for single crystalline SnSe [24]. In Fig. 2c, the temperature-dependent electrical conductivity of single crystalline SnSe is shown for the semiconductor range (30–100 K) and for the metallic range (>100–300 K). Above 100 K, the grown SnSe crystals exhibited metallic transport behavior, consistent with previous observations [24].

**Fig. 3:**
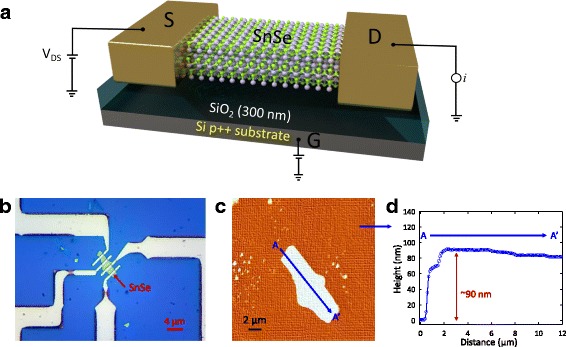
**a** Schematic of a mechanically exfoliated SnSe nanoflake FET on a SiO_2_/p^++^ Si substrate. **b** Optical image of a fabricated SnSe nanoflake FET that was used for electrical transport measurements. **c** AFM image of a SnSe nanoflake on a SiO_2_/Si substrate. **d** AFM height profile of a SnSe nanoflake, for estimating the thickness of, and fabricating FET devices

**Fig. 4:**
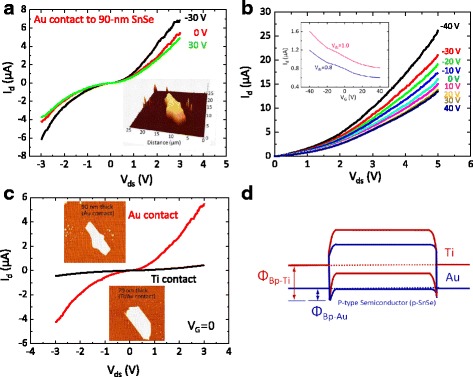
**a** Drain current (*I*_d_) as a function of applied source-drain voltage (*V*_ds_), for the gate voltages (*V*_g_) of −30, 0, and 30 V, for a 90-nm-thick SnSe nanoflake FET, at room temperature. **b** *I*_d_ vs. *V*_ds_ for *V*_g_ ranging from −40–40 V in steps of 10 V, for the 90-nm-thick SnSe nanoflake FET. The *inset* shows *I*_d_ vs. *V*_g_ for *V*_ds_ of 0.8 and 1.0 V, measured at room temperature. **c** *I*_d_ vs. *V*_ds_ without biasing *V*_g_ (=0) for Au and Ti contacts on a SnSe nanoflake FET. The *inset* shows an AFM scanned image of SnSe nanoflakes. **d** Schematics of the energy band diagrams of two metals, Au and Ti, on p-type SnSe semiconductors

SnSe FET devices were fabricated as follows. First, SnSe nanoflakes were mechanically exfoliated onto a 300-nm-thick SiO_2_/p^++^ Si substrate from single SnSe crystals, easily cleaved in the (100) plane using the well-known scotch tape method (Fig. 1e) [26, 27, 34]. Measurements of electrical transport in as-prepared individual SnSe nanoflakes FETs were performed at room temperature in the back-gated FETs configuration. Figure 3a schematically shows SnSe nanoflake-based FET devices with a large-area back gate. In this study, two SnSe nanoflakes (70- and 90-nm-thick SnSe nanoflakes) were prepared on the SiO_2_/Si substrate. The SnSe FETs were fabricated using the standard electron-beam lithography method followed by two types of metallization, i.e., Au (thickness, 100 nm) and Ti/Au (thicknesses, 10/100 nm) were considered as ohmic contacts on SnSe nanoflakes. Before the metallization process, buffered oxide etching (BOE) was performed to remove the polymer and oxide residues on the nanoflakes’ surfaces. Figure 3b shows an optical image of a SnSe nanoflake FET with a device thickness of 90 nm. The thicknesses of the SnSe nanoflakes were measured using an atomic force microscope (AFM) at room temperature (Fig. 3c–d). As shown in Fig. 3b and inset of Fig. 2b, the fabricated 90-nm-thick SnSe FET had the channel length (*L*) of 5 μm and width (*W*) of 4.71 μm, while for the 70-nm-thick SnSe FET *L* was 5 μm and *W* was 6 μm. All of the current–voltage (*I*-*V*) characteristics were measured using a semiconductor parameter analyzer (HP 4155C, Agilent Technologies, USA) on an electrically shielded probe station at room temperature. Figure 4a shows the drain current (*I*_d_) as a function of the gate voltage (*V*_g_), for the 90-nm-thick SnSe nanoflake, for the source-drain voltages (*V*_ds_) of −30, 0, and 30 V, at room temperature, indicating a clear p-type semiconductor behavior, which is mainly attributed to the Sn vacancies, as reported previously [15, 16, 22, 24, 35–39]. The result in Fig. 4a implies that metallic AU with its high work function is expected to form weak ohmic contacts on SnSe nanoflakes, indicating a lower Schottky barrier for the conduction band of SnSe nanoflakes. A more detailed discussion, for work functions of different metals, will be provided later. Figure 4b shows *I*_d_ vs. *V*_ds_ for different *V*_g_, ranging from −40–40 V, in steps of 10 V. From Fig. 4b, the hole mobility (*μ*_p_) is determined to be ~2.7 cm^2^/V s, obtained from *μ*p = *t*_*m*_[*L* ⁄ (*WC*_ox_ *V*ds)], where *t*_m_ is the trans-conductance (=*dI*_d_/*dV*_g_ = 2.89 × 10^− 8^ *A*/*V*), *L* is the length (~5.1 μm), *W* is the width (~4.75 μm), *V*_ds_ is the drain-source voltage (~1 V) of the SnSe FET, and *C*_ox_ (=*ε*_*r*_ *ε*_0_/*d* = 11.5 nF/cm^2^) with *ε*_r_ (the dielectric constant) of 3.9 and *d* (the thickness of the oxide layer) of 300 nm is the capacitance per unit area of the back-gated SnSe nanoflake FET. The evaluated hole mobility of the mechanically exfoliated SnSe nanoflake FETs is much smaller than that of epitaxial SnSe thin films (~60 cm^2^/V s) prepared by pulsed laser deposition on MgO substrates using Se-rich targets [40]. However, the value obtained here is ~1.8 times larger than that obtained for single-crystal SnSe nanoplates (~1.5 cm^2^/V s) [33]. Such a relatively low hole mobility can be attributed to a strong phonon scattering owing to the Sn vacancies on the SnSe surface [18, 36, 41, 42] and a relatively high Schottky barrier at the Au metal-SnSe nanoflake interface.

In addition, we observed a weak gate tuning of conductance in the depletion region of the *I*_d_ vs. *V*_ds_ curve (Fig. 4c) and a low current on/off ratio (~2 at *V*_ds_ of 1 V, insets of Fig. 4c) in the p-SnSe nanoflake FET with Au metal contacts. A similar behavior was reported for other two-dimensional (2D) semiconducting materials with a similar thickness, including SnS FETs (thickness, ~50–80 nm) [43], ~15.8-nm-thick SnSe nanoplates [33], ~80-nm-thick MoS_2_ [44], and ~84-nm-thick SnSe_2_ [26]. These behaviors can be explained by the finite carrier screening length effect owing to the existence of a superficial conductive surface layer in FET devices with thicknesses larger than the screening length $\left(\sqrt{\varepsilon {K}_B T}{e}^2 p\right)$, where *ε*, *K*_B_, and *p* are the dielectric constant of the semiconductor, Boltzmann’s constant, and hole carrier density, respectively, [43].

Metal contacts importantly determine the characteristics of 2D FET devices [45]. To determine the effect of metal’s work functions on SnSe, we considered Au (work function, ~5.1 eV) and Ti (work function, ~4.3 eV) as metal contacts on SnSe nanoflakes. Figure 4c shows typical *I*_d_ vs. *V*_ds_ curves without gate modulation (*V*_g_ = 0) for SnSe nanoflake FETs with Au and Ti contacts, indicating a higher overall resistance for Ti (~15.4 MΩ) compared with that for Au (~0.56 MΩ). Thus, the Schottky barrier at the metal-SnSe interface is higher for Ti (Fig. 4c). This behavior is always observed on all the SnSe FET with Ti contacts. As shown in Fig. 4d, the height of the Schottky barrier for holes increases as the work function of the metal decreases. Thus, metals such as Pd, Au, and Pt, with large work functions, can be suitable as ohmic contacts on p-SnSe nanoflake FETs because for these metals, the height of the Schottky barrier for injection of holes will be lower. Contact resistance should be measured for additional metals, to determine their suitability as metal contacts on SnSe nanoflake. This issue is currently being addressed using the transfer length method.

## Conclusions

In summary, multi-layer SnSe nanoflakes were grown, exfoliated, and characterized for SnSe FET channels with a back-gated FET structure on SiO_2_/Si substrates. Electrical transport measurements demonstrated that multi-layer SnSe nanoflakes with Au metal contacts exhibit p-type semiconductor characteristics with a relatively low Schottky barrier and low contact resistance on exfoliated SnSe nanoflake FETs. In addition, we emphasize that this study is the first one to report mechanically exfoliated SnSe nanoflake-based FETs and we are confident that our SnSe nanoflake FETs are very promising for 2D electrical devices as well as for energy harvesting applications, including future generation of thermoelectricity.
